# Slit2 inhibits SRC-PI3K signaling pathway, regulates osteoclast differentiation of macrophages and reduces bone resorption

**DOI:** 10.3389/fcell.2025.1632882

**Published:** 2025-08-26

**Authors:** Qing Ge, Yaokun Zou, Huizhi Deng, Zhe Yang, Linhu Ge, Jieying Huang, Jianqiang Cai, Liping Wang

**Affiliations:** ^1^ Department of Oral Implantology, School and Hospital of Stomatology, Guangdong Engineering Research Center of Oral Restoration and Reconstruction and Guangzhou Key Laboratory of Basic and Applied Research of Oral Regenerative Medicine, Guangzhou Medical University, Guangzhou, Guangdong, China; ^2^ Department of Oral Implantology, Suzhou Stomatological Hospital, Suzhou, China

**Keywords:** Slit2, osteoporosis, osteoclasts, Src, PI3K/AKT signaling

## Abstract

Osteoporosis is a metabolic disorder characterized by increased bone resorption and decreased bone formation. As a well-characterized axon guidance molecule, Slit2 contributes to central nervous system regulation by modulating intracellular signaling cascades and is expressed in multiple tissues, including the nervous system and the kidneys. However, limited research has explored the role of Slit2 in bone metabolism, and its precise regulatory mechanisms remain unclear. In this study, we established an aging-induced osteoporosis model using wild-type (WT) and Slit2-transgenic (Slit2-Tg) mice, as well as an estrogen-deficient ovariectomy-induced osteoporosis model. Our findings demonstrate that Slit2 attenuates bone loss and suppresses osteoclast differentiation under osteoporotic conditions. *In vitro* osteoclast differentiation assays further confirmed that Slit2 regulates osteoclast-associated marker expression and inhibits the differentiation of bone marrow-derived monocytes into osteoclasts. Mechanistically, RNA sequencing, Gene Ontology pathway enrichment analysis, and Western blotting revealed that Src and PI3K/AKT signaling pathways mediate the regulatory effects of Slit2 on bone metabolism. These findings suggest that Slit2 inhibits macrophage-to-osteoclast differentiation and attenuates bone resorption by downregulating Src expression in macrophages and suppressing the PI3K/AKT signaling pathway. This study provides novel insights into the therapeutic potential of Slit2 as a target for osteoporosis treatment.

## 1 Introduction

Osteoporosis (OP) is one of the most prevalent metabolic bone diseases, characterized by low bone mass, deterioration of bone microarchitecture, increased bone fragility, and a heightened risk of fractures (Aibar-Almazán et al., 2022). As menopause or aging progresses, individuals experience progressive bone loss, leading to the increasing global prevalence of osteoporosis, which is particularly common among postmenopausal women and the elderly (Sözen et al., 2017). Bone undergoes continuous remodeling, allowing for constant renewal and adaptation to maintain dynamic balance throughout adulthood. This process is primarily regulated by the balance between bone formation by osteoblasts (OBs) and bone resorption by osteoclasts (OCs). Disruptions in this balance, where OB and OC activity become dysregulated, lead to bone loss, which forms the basis of osteoporosis pathogenesis (Da et al., 2021). Therefore, identifying key molecules involved in bone homeostasis may facilitate the development of targeted therapies, offering meaningful advancements in osteoporosis treatment.

Slit2 is a secreted protein expressed in various tissues, including the nervous system, kidneys, lungs, heart, and immune cells (Yuan et al., 1999; Chaturvedi and Robinson, 2015; Sherchan et al., 2016; Zhao et al., 2022). It functions by binding to specific Robo receptors, thereby regulating diverse cellular processes (Wang et al., 2022). Previous studies have primarily focused on its roles in angiogenesis, neuronal migration, and cancer cell motility (Li et al., 1999; Yuen and Robinson, 2013; Yin and Zhao, 2022). In the early 21st century, studies found that Slit2 is expressed in bone tissue and inhibits osteoblast differentiation in rat MC3T3 cells (Sun et al., 2009). However, another study found that Slit2 inhibits bone resorption by reducing osteoclast formation through the suppression of Cdc42 activity, while having no biological effect on osteoblasts (Park et al., 2019). Therefore, the role and specific mechanism of Slit2 in bone metabolism remain unclear.

Among the mechanisms implicated in OP pathogenesis, the PI3K/AKT signaling pathway plays a crucial role in bone homeostasis. Studies have shown that this pathway promotes osteoblast proliferation and differentiation, thereby helping to prevent osteoporosis (Xi et al., 2015). Moreover, other studies have found that the PI3K/AKT signaling pathway enhances osteoclast proliferation and differentiation and plays a crucial role in osteoclast survival. Macrophage colony-stimulating factor (M-CSF) binds to the CSF-1R receptor on osteoclast precursor cells, activating tyrosine kinase Src, as well as PI3K and AKT, thereby promoting osteoclast proliferation and differentiation (Bartell et al., 2014; Lu et al., 2020). Src is an upstream component of the PI3K/AKT signaling pathway. Studies have shown that Slit2 binds to Robo1 and modulates Src kinase activity, facilitating its tyrosine phosphorylation (Zhang et al., 2015). Certain tyrosine kinases play a critical role in osteoclast formation and bone resorption (Zhao et al., 2018). However, the relationship between Slit2 and Src, and the PI3K/AKT pathway, during osteoporosis requires further investigation.

In this study, we used Slit2 transgenic (Slit2-Tg) mice to investigate the role of Slit2 in osteoclastogenesis and bone mass regulation. Our results indicate that Slit2 reduces osteoclast numbers and suppresses bone resorption. Furthermore, Slit2-Tg mice exhibited significantly lower bone loss in both aging-induced and ovariectomy-induced osteoporosis models. Mechanistically, we elucidated that Slit2 inhibits PI3K/AKT signaling, with Src playing a critical role in this regulation. Collectively, these findings provide new insights into the role of Slit2 in bone metabolism and highlight its potential as a therapeutic target for OP.

## 2 Materials and methods

### 2.1 Animals

Wild-type (WT) C57BL/6 mice used in this study were obtained from the Guangdong Animal Experiment Center. Slit2-Tg overexpression mice were obtained from Vascular Biology Research Institute, School of Life Sciences and Biopharmaceutics, Guangdong Pharmaceutical University, Guangzhou 510006, China. Slit2-Tg mice were generated according to the previously reported protocol (Yang et al., 2010). All the mice used in the experiments, including females and aged 8 weeks, 24 weeks, 48 weeks, and 70 weeks, were specific pathogen free (SPF) grade. All the mice were confirmed to be in healthy and normal physiological conditions prior to experimentation. The Institutional Laboratory Animal Care and Use Committee of Guangzhou Medical University approved this study (GY 2021-068).

### 2.2 Genotyping of genetically modified mice

Mice selected for genotyping were identified by notching a single ear and recording the corresponding ID number. Approximately 0.5–0.8 cm of tail tissue was excised and placed into a sterile EP tube containing 180 μL of 50 mmol/L sodium hydroxide (NaOH) solution, ensuring complete submersion of the tissue. Samples were incubated in a metal dry bath at 100 °C for 60 min to lyse the cells. After cooling to room temperature, 20 μL of 1 mol/L hydrochloric acid (HCl) was added for neutralization. The mixture was thoroughly vortexed, and the resulting supernatant was collected and used as the genomic DNA template.

Polymerase chain reaction (PCR) was performed to amplify the target gene. The PCR protocol included an initial denaturation at 94°C for 4 min, followed by 35 cycles of denaturation at 94°C for 45 s, annealing at 60°C for 45 s, and extension at 72°C for 45 s. A final extension step at 72°C for 10 min was applied, and the PCR products were stored at 4°C.

For agarose gel electrophoresis, a 1 × TAE buffer was used to prepare the agarose gel. After melting, a nucleic acid stain was added to the gel solution prior to casting. Once solidified, combs were inserted to form wells. PCR products were loaded into the wells and subjected to electrophoresis. Gel bands were visualized using a gel imaging system to assess amplification results, thereby confirming whether the genotype of each mouse met the experimental requirements.

### 2.3 Micro-CT analysis

The femur tissues of the mice were fixed in 4% paraformaldehyde at room temperature for 24 h. The fixed femur tissues were scanned by SkyScan1172 (Bruker-Micro-CT, Kontich, Belgium) using 60 kV device voltage 100 uA current, and set the scanning layer thickness to 10 μm and the baffle thickness to 0.5 mm. The NRECON and CTVOX software was used for the 3D reconstruction of the scanned images. The 3D images were then examined using Dataviewer software for alignment and registration. The CTan software was utilized for 2D/3D femoral image reconstruction and analysis to assess bone microarchitecture.

### 2.4 Histological evaluation

The femoral tissues were fixed in 4% paraformaldehyde (PFA) for 2 days and subsequently decalcified in EDTA decalcification solution for 21 days. Subsequently, 3 μm-thick bone tissue sections were cut along the coronal plane. The decalcified sections were further processed for deparaffinization, hematoxylin and eosin (H&E) staining, and dehydration.

### 2.5 Tartrate-Resistant Acid Phosphatase (TRAP) staining

Tissue sections were stained using a Tartrate-Resistant Acid Phosphatase (TRAP) staining kit (G1050, Servicebio, Wuhan, China). According to the manufacturer’s protocol, the TRAP staining solution was prepared and applied to the sections, followed by incubation in the dark until distinct osteoclasts were observed under the microscope. For TRAP staining of osteoclasts cultured in 48-well plates, cells were fixed with 4% paraformaldehyde, and the prepared TRAP staining solution was added to each well to cover all cells. The plates were then incubated at 37 °C in the dark until distinct osteoclasts were observed under the microscope.

### 2.6 Immunohistochemistry

Tissue sections were incubated with citrate sodium antigen retrieval solution (EDTA, Solarbio) in a pressure cooker for 10 min to restore antigenicity. After outlining the tissue sections with an immunohistochemical pen, endogenous peroxidase blocking solution was applied, and the sections were incubated in a humid chamber at 37 °C for 30 min. After removing the blocking solution, primary antibodies were added, and the sections were incubated overnight at 4 °C, followed by returning to room temperature. Corresponding secondary antibodies were then applied, and the sections were incubated at 37°C for 1 h. An appropriate amount of DAB chromogenic solution was added, and the sections were observed under a microscope. Images of the tissue sections were captured using an optical microscope, and immunohistochemical staining cells were counted.

### 2.7 Cell culture

Primary mouse bone marrow cells were obtained by flushing the femurs and tibias of mice. The collected cells were cultured in complete medium (DMEM supplemented with 10% FBS and 1% antibiotics: 100 U/mL streptomycin and 100 U/mL penicillin). After 24 h, non-adherent bone marrow-derived monocytes (BMMs) were isolated and cultured in complete medium supplemented with 30 ng/mL macrophage colony-stimulating factor (M-CSF (CB34, NovoProtein)). The medium was replaced every 48 h, and bone marrow-derived macrophages (BMDMs) were collected on day 3.

For osteoclast differentiation, BMDMs were seeded at 3 × 10^4^ cells per well in 48-well plates and cultured in differentiation medium containing 100 ng/mL receptor activator of nuclear factor-κB ligand (RANKL (CJ94, NovoProtein)) and 30 ng/mL M-CSF. The medium was refreshed every 2 days, and osteoclasts were obtained by day 6.

Macrophages were treated with 10 μM Src activator Tolimidone (MedChemExpress)) prior to RNA-seq and cytokine profiling (Zhuang et al., 2020).

### 2.8 Osteogenic differentiation induction and ALP staining

Primary bone marrow stromal cells (BMSCs) were isolated from 8-week-old WT and Slit2-Tg mice by flushing femurs and tibias. BMSCs were cultured in 48-well plates at 5 × 10^5^ cells/well in complete DMEM (DMEM contained 10% FBS, 100 U/mL penicillin, and 100 μg/mL streptomycin) at 37 °C for 24 h. The culture medium was subsequently changed to osteogenic differentiation medium (OM) containing DMEM (glucose: 1,000 mg/L), 10 %FBS, 100 IU/mL penicillin, 100 μg/mL streptomycin, 0.1 μM dexamethasone (DXMS), 10 mM β-glycerol phosphate, and 50 μM ascorbic acid for 7 days. The medium was replaced every 3 days. After 7 days of culture in OM, the cells were washed with PBS and subsequently fixed for 15 min with formaldehyde solution. Then, the cells were stained using a BCIP/NBT ALP kit (P0321S, Beyotime) according to the manufacturer’s instructions. The cells were observed and photographed with an optical microscope.

### 2.9 Immunofluorescence staining

Bone marrow-derived macrophages (BMMs) from WT mice and Slit2-Tg mice were seeded on 24-well chamber slides at a density of 1 × 10^5^ cells per well. After 24 h, the medium was replaced with complete medium containing M-CSF and RANKL, and the medium was changed every 2 days. After 5 days, the medium was removed, and the cells were fixed with 4% paraformaldehyde. Permeabilization was performed using 0.2% Triton X-100 for 20 min, followed by blocking with blocking buffer. After blocking, the cells were incubated with primary antibody at 4 °C overnight. Subsequently, the cells were incubated with Phalloidin (Solarbio) for 1 h, followed by incubation with the corresponding secondary antibody at 37 °C in the dark for 1 h. Finally, the cells were stained with Hoechst in the dark for 10 min. The chamber slides were then observed under a confocal laser scanning microscope.

### 2.10 RT-qPCR

Femoral tissues from WT and Slit2-Tg mice were homogenized in a liquid nitrogen mortar, and total RNA was extracted using Trizol. Total RNA from osteoclasts was isolated using Trizol. Reverse transcription of total RNA was performed using Takara PrimeScriptTM RT Master Mix. RT-qPCR for specific genes was conducted using Takara TB-Green Premix Ex Taq on a LightCycler RT-qPCR system (LC-480 II, Roche). The primers used in this study are listed in Table 1.

**TABLE 1:** Primer sequence.

Gene name	Forward sequence (5’->3′)	Reversed sequence (5’->3′)
*Slit2*	CCC TCC GGA TCC TTT ACC TGT TCA AGG TCC T	TGG AGA GAG CTC ACA GAA CAA GCC ACT GTA
*Trap*	AGC CAA GGA CTA C	CAT AGC CCA CAC CGT TCT C
*Nfatc1*	GGA GAG TCC GAG AAT CGA GAT	TTG CAG CTA GGA AGT ACG TCT
*Ctsk*	GAA GAC TCA CCA GAA GCA G	TCC AGG TTA TGG GCA GAG ATT
*Gapdh*	TGT GTC CGT GGA TCT G	TTG CTG TTG AAG TCG CAG GA

### 2.11 Western blot analysis

BMMs were seeded in 6-well plates at a density of 5 ×10^5^ cells per well. After inducing the cells into osteoclasts as described previously, cell lysates were prepared using lysis buffer (P0013B, Beyotime). Total proteins were separated by SDS-PAGE and transferred onto polyvinylidene difluoride (PVDF) membranes (Immobilon). The membranes were blocked with blocking buffer (P0252, Beyotime) at room temperature for 30 min and incubated overnight at 4 °C with primary antibodies: anti-NFACT1 (1:1000, CST), anti-TRAP (1:1000, ABclonal), anti-CTSK(1:1000, CST), anti-PI3K (1:1000, CST), anti-P-PI3K (1:1000, CST), anti-SRC (1:1000, CST),anti-P-SRC (1:1000,CST), anti-GAPDH (1:100000, Proteintech). After washing three times with TBST, the membranes were incubated with horseradish enzyme-labeled secondary antibodies at room temperature for 1 h. The membranes were washed three times with TBST. Immunoreactive protein bands were detected using an enhanced chemiluminescence detection system (BLT GelView 6000 Pro, BioLight, Guangdong, China).

### 2.12 Enzyme-linked immunosorbent assay (ELISA)

Blood was collected from the orbital venous plexus of mice and allowed to stand at room temperature for 1 h. After centrifugation at 1200 rpm for 15 min to separate plasma from serum, the supernatant was transferred to EP tubes. The expression level of the osteoclast marker CTX1 in mouse serum was detected using a commercially available ELISA kit (Elabscience, China), followed by statistical analysis.

### 2.13 RNA sequencing

BMMs were extracted from WT and Slit2-Tg mice, and total RNA of osteoclasts was isolated using Trizol after osteoclast induction. Library construction was performed using the Illumina TruseqTM RNA Sample Prep Kit (Illumina, USA). The constructed libraries were quantified using Qubit and subjected to quality control. Qualified libraries were sequenced on the Illumina Hiseq 2000 platform. Data quality control was performed using fastp, and valid data were analyzed for principal component analysis (PCA), differential expression analysis, cluster analysis, Gene Ontology (GO) functional annotation and enrichment analysis, as well as KEGG pathway annotation and enrichment analysis.

### 2.14 Statistical analyses

All data were presented as mean ± standard deviation (SD). An unpaired, nonparametric Mann-Whitney test or a two-tailed unpaired t-test was used to determine differences between the two groups. For multiple comparisons of groups, a one-way analysis of variance (ANOVA) was performed followed by Bonferroni’s multiple comparison test. Data were analyzed with GraphPad Prism 8.0 statistical software. Values were considered significantly different if p < 0.5.

## 3 Results

### 3.1 Slit2 overexpression alleviates bone loss and enhances bone strength in both young and aged mice

**FIGURE 1:**
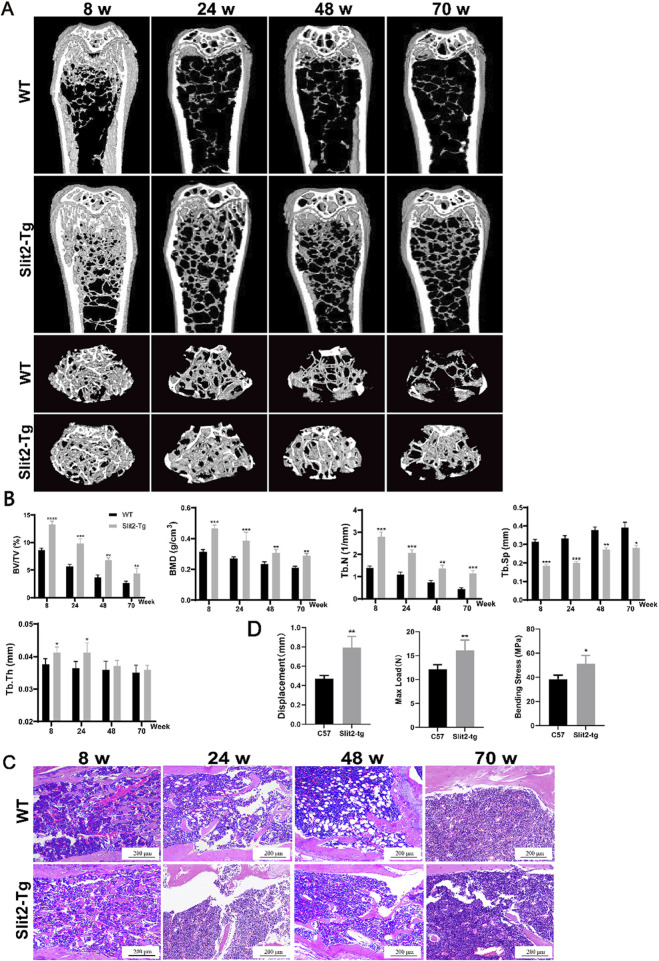
Slit2 overexpression alleviates bone loss in both young and aged mice. **(A)** Micro-CT three-dimensional reconstruction images of femur tissues from WT and Slit2-Tg mice at different ages. **(B)** H&E staining results of femur tissues of WT mice and Slit2-Tg mice of different weeks of age, with a scale of 200 μm. **(C)** CTan software analysis results of femur bone status of WT mice and Slit2-Tg mice of different weeks of age (n = 5). **(D)** Experimental results of three-point femur bending in two groups of mice aged 24 weeks (n = 4). Data are presented as mean ± SD. Significant differences among groups are indicated as *p < 0.05, **p < 0.01, ***p < 0.001, and ****p < 0.0001. BV/TV: relative bone volume; BMD: bone mineral density; Tb.N: trabecular number; Tb.Sp: trabecular separation; Tb.Th: trabecular thickness; Displacement: displacement of femur at fracture, Max Load: maximum load at fracture, Bending Stress: bending stress at fracture.

To investigate the bone quality of Slit2-Tg mice compared to WT mice in age-related osteoporosis, we established an age-related osteoporosis model using Slit2-Tg and WT mice. Mice were naturally raised under laboratory conditions and sacrificed at 8, 24, 48, and 70 weeks of age, followed by Micro-CT scanning of the femoral samples. Detailed observation and comparison of femurs from Slit2-Tg and WT mice at each stage using Micro-CT revealed that Slit2-Tg mice exhibited superior bone density and bone performance compared to WT mice across all age groups (Figure 1A). Analysis of the Micro-CT data using CTan software showed that Slit2-Tg mice had significantly higher bone mineral density (BMD), relative bone volume (BV/TV), trabecular number (Tb.N), and trabecular thickness (Tb.Th) in the femoral metaphysis, while trabecular separation (Tb.Sp) was significantly lower than in WT mice (Figure 1B). These results indicate that the femoral microstructure of Slit2-Tg mice is superior to that of WT mice, with consistent trends observed across all age groups. To further evaluate the stress resistance of femurs in both groups, additional experiments were performed. H&E staining of the femoral metaphysis demonstrated that Slit2-Tg mice had denser trabecular bone and a more compact trabecular surface structure compared to WT mice at different ages (Figure 1C), suggesting enhanced biomechanical properties and improved stress resistance in Slit2-Tg mice. Three-point bending tests further revealed that femurs from Slit2-Tg mice exhibited greater displacement, maximum load, and bending stress at fracture compared to WT mice (Figure 1D), indicating that Slit2-Tg mice possess stronger bones capable of withstanding greater stress and exhibiting higher bone density.

### 3.2 Slit2-Tg mice exhibit superior bone quality in ovariectomy-induced osteoporosis models

**FIGURE 2:**
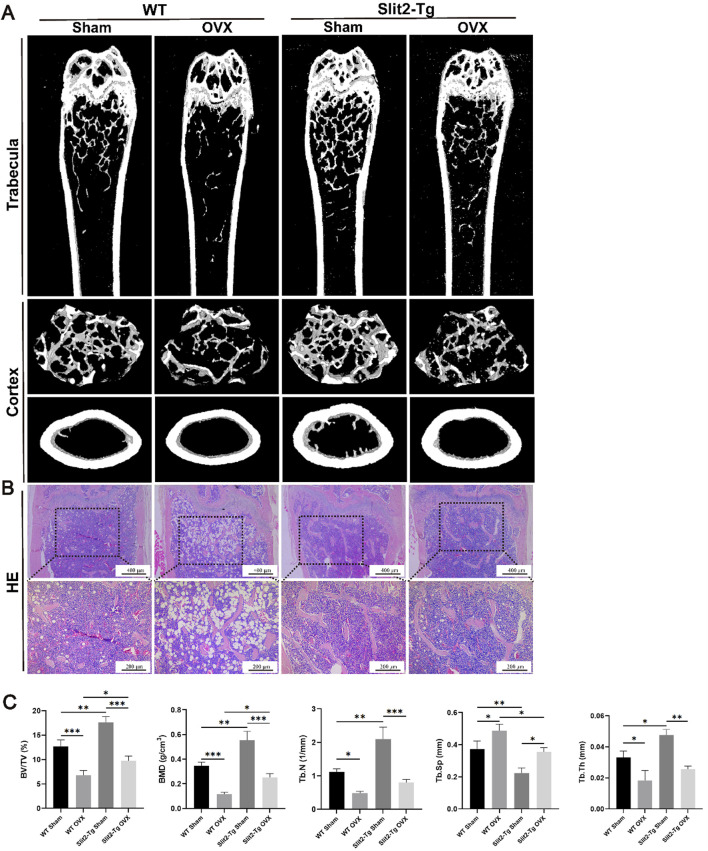
Comparative analysis of femoral bone properties between WT and Slit2-Tg mice in the OVX-induced osteoporosis model. **(A)** Micro-CT three-dimensional reconstruction images of femur tissues from sham-operated and OVX-induced WT and Slit2-Tg mice. **(B)** Results of H&E staining of femur tissues of WT mice and Slit2-Tg mice in ovarian castration osteoporosis model. **(C)** Results of CTan software analysis of femur bone conditions of WT mice and Slit2-Tg mice in ovarian castration osteoporosis model (n = 5). Data are presented as mean ± SD. Significant differences among groups are indicated as *p < 0.05, **p < 0.01 and ***p < 0.001. BV/TV: relative bone volume; BMD: bone mineral density; Tb.N: trabecular number; Tb.Sp: trabecular separation; Tb.Th: trabecular thickness.

To further validate the role of Slit2 in other types of osteoporosis, we established ovariectomy (OVX)-induced osteoporosis models using 8-week-old WT and Slit2-Tg mice. Mice were sacrificed at 12 weeks post-surgery, and femoral metaphysis samples were subsequently collected for analysis via Micro-CT scanning and H&E staining. Micro-CT analysis demonstrated that within the OVX groups, Slit2-Tg mice exhibited significantly higher bone mineral density (BMD) than WT mice (Figure 2A). Quantitative analysis using CTan software revealed that OVX Slit2-Tg mice had increased relative bone volume (BV/TV) and trabecular separation (Tb.Sp) compared to OVX WT mice, whereas no significant differences were observed in trabecular number (Tb.N) or trabecular thickness (Tb.Th) (Figure 2C). These findings indicate that Slit2-Tg mice maintain a more preserved trabecular bone structure in the OVX model, consistent with results observed in the aging-induced osteoporosis model. H&E staining revealed that Slit2 plays a critical role in maintaining bone homeostasis in the OVX-induced osteoporosis model (Figure 2B).

### 3.3 Slit2 is highly expressed in the femur of Slit2-Tg mice and Slit2-Tg mice exhibit a reduced number of osteoclasts in femurs compared to wild-type controls

**FIGURE 3:**
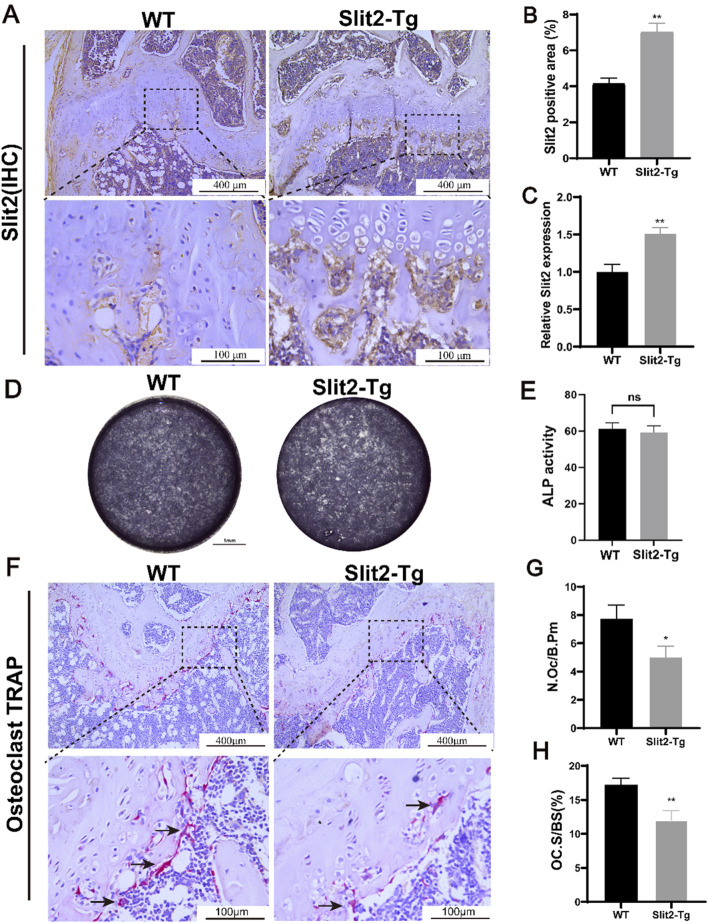
Slit2-Tg mice demonstrate reduced osteoclast numbers in femurs compared to WT mice. **(A)** Immunohistochemical staining of Slit2 in the epiphysis of femoral shaft in 8-week-old WT mice and Slit2-Tg mice, with scales of 400 μm and 100 μm, respectively. **(B)** Semi-quantitative immunohistochemical analysis of Slit2 in femur tissues of WT mice and Slit2-Tg mice (n = 5). **(C)** Slit2 mRNA expression in femur tissues of WT mice and Slit2-Tg mice (n = 5). **(D)** 7 days Alkaline phosphatase (ALP) staining in 8-week-old WT and Slit2-Tg mice BMSC. **(E)** Statistical analysis of ALP activity at day 7 (n = 4). **(F)** TRAP staining results of femur tissue of 70-week-old WT mice and Slit2-Tg mice with a scale of 100 μm. **(G)** The number of osteoclasts per unit of bone surface in femoral tissue sections of 70-week-old WT mice and Slit2-Tg mice (n = 6). **(H)** The percentage of osteoclast area per unit bone surface in femoral tissue of 70-week-old WT and Slit2-Tg mice (n = 6). Data are presented as mean ± SD. Significant differences among groups are indicated as *p < 0.05, **p < 0.01.

After the construction and expansion of the Slit2 transgenic (Slit2-Tg) mouse line, genotyping was performed prior to each experiment to prevent off-target effects that may compromise the accuracy of experimental results (Supplementary Figure S1). Following genotyping, to determine the expression and localization of Slit2 in femoral bone tissue of Slit2-Tg mice, we performed histological analysis on femoral sections from 8-week-old WT and Slit2-Tg mice. Eight-week-old mice were selected because this age represents a physiologically mature stage with active bone remodeling but minimal age-related degeneration, allowing for a clear assessment of Slit2 expression patterns under steady-state conditions. Immunohistochemical (IHC) staining revealed that, compared to WT mice, Slit2-Tg mice exhibited more intense staining in the femoral metaphysis, indicating significantly higher Slit2 expression levels (Figures 3A,B). Total RNA was extracted from the femurs of both groups, followed by reverse transcription for further molecular analysis. RT-qPCR results indicated that Slit2 mRNA levels were significantly increased in the femur tissue of Slit2-Tg mice compared to WT mice (Figure 3C). To further investigate the specific role of Slit2 in age-related osteoporosis, we isolated bone marrow stromal cells (BMSCs) from 8-week-old WT and Slit2-Tg mice. After 7 days of osteogenic induction, alkaline phosphatase (ALP) staining revealed no significant difference in ALP activity between WT and Slit2-Tg mice (Figures 3D,E), suggesting that the osteoprotective effect of Slit2-Tg mice in OP may not be mediated by enhanced osteogenesis. Subsequently, TRAP staining was performed on femur sections from 70-week-old WT and Slit2-Tg mice. This later time point was chosen to assess osteoclast activity in a natural aging context, as 70-week-old mice typically exhibit an established osteoporotic phenotype, providing a suitable model for evaluating the regulatory effects of Slit2 on osteoclastogenesis under osteoporotic conditions. The results demonstrated that, compared to WT mice, Slit2-Tg mice exhibited a significantly lower number of osteoclasts per unit of bone surface (N.Oc/B.Pm) and a reduced percentage of osteoclast area per unit bone surface (OC. S/BS, %) (Figures 3F–H). These results indicate that the number of mature osteoclasts (defined as those with ≥3 nucei) was markedly lower in Slit2-Tg mice, with osteoclasts also displaying smaller cell sizes compared to those in WT mice.

### 3.4 Osteoclast differentiation is inhibited in bone marrow-derived macrophages from Slit2-Tg mice

**FIGURE 4:**
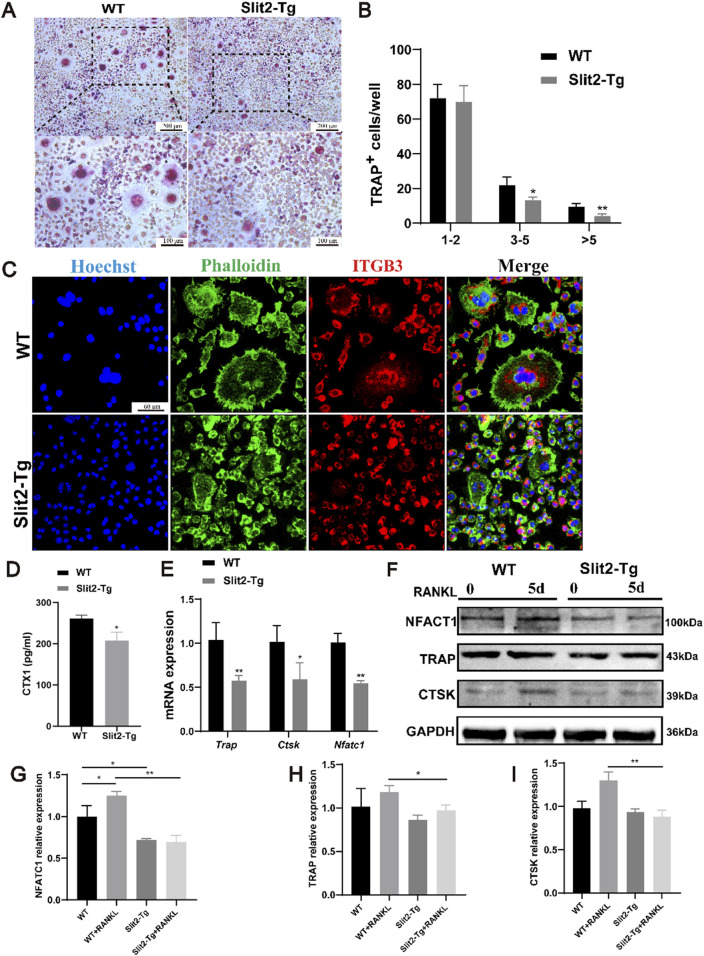
Osteoclast differentiation is inhibited in bone marrow-derived macrophages from Slit2-Tg mice. **(A)** TRAP staining results of 8-week-old WT mice and Slit2-Tg mice after 5 days of osteoclast differentiation induction with M-CSF and RANKL and M-CSF medium. **(B)** TRAP staining statistics of BMMs in WT mice and Slit2-Tg mice after osteoclast differentiation induction (n = 5). **(C)** The BMMs of 8-week-old WT mice and Slit2-Tg mice were induced by osteoclast differentiation using M-CSF and RANKL medium for 5 days, and the results of immunofluorescence staining were 60 μm. **(D)** The serum CTX1 content of 70-week-old WT mice and Slit2-Tg mice (n = 5). **(E)** Gene expression in BMMs of WT and Slit2-Tg treated with RANKL (100 ng/mL) and M-CSF (30 ng/mL) for 5 days (n = 3). **(F–I)** Protein expression in BMMs of WT and Slit2-Tg treated with RANKL (100 ng/mL) and M-CSF (30 ng/mL) for 0 or 5 days (n = 3). The significant difference among the groups, *p < 0.05, **p < 0.01 and ***p < 0.001.

To investigate whether Slit2 overexpression directly affects osteoclast differentiation in macrophages, bone marrow-derived monocytes (BMMs) were isolated from 8-week-old WT and Slit2-Tg mice. The cells were cultured in osteoclastogenic medium supplemented with M-CSF (30 ng/mL) and RANKL (100 ng/mL) for 5 days. TRAP staining was performed post-induction to assess osteoclast differentiation across experimental groups, there by elucidating the specific role of Slit2 in macrophage osteoclastogenesis. TRAP staining demonstrated that BMMs from Slit2-Tg mice exhibited significantly suppressed fusion capacity during osteoclast differentiation compared to WT mice (Figures 4A,B). Consistently, immunofluorescence staining revealed significantly fewer osteoclasts derived from Slit2-Tg BMMs after induction compared to WT counterparts (Figure 4C) leading to the formation of smaller and fewer osteoclasts. To systemically investigate the relationship between Slit2 and osteoclast differentiation at the organismal level, serum samples from 70-week-old WT and Slit2-Tg mice were analyzed via ELISA. The results demonstrated that serum levels of C-terminal telopeptide of type I collagen (CTX-1), a bone resorption marker, were significantly lower in 70-week-old Slit2-Tg mice compared to age-matched WT controls (Figure 4D). This suggests that Slit2 overexpression may lead to a reduction in bone resorption markers, indicating potential suppression of bone resorption activity. *In vitro*, BMMs isolated from WT and Slit2-Tg mice were induced to differentiate into osteoclasts, and the expression of osteoclast-related markers was subsequently analyzed at the molecular level. RT-qPCR results revealed that, compared to WT-derived BMMs, mRNA expression levels of osteoclast-associated markers, including *Trap*, *Ctsk*, and *Nfatc1*, were significantly downregulated in Slit2-Tg-derived BMMs (Figure 4E). To further validate the findings, Western blot analysis revealed that CTSK and NFATC1 protein levels were markedly reduced in BMMs derived from Slit2-Tg mice compared to those from WT mice. The expression of the osteoclast-associated marker TRAP also showed difference between the two groups (Figures 4F–I). These findings suggest that Slit2 overexpression may inhibit osteoclast differentiation by downregulating the expression of key osteoclast-associated genes.

### 3.5 Slit2 overexpression inhibits the PI3K/AKT signaling pathway

**FIGURE 5:**
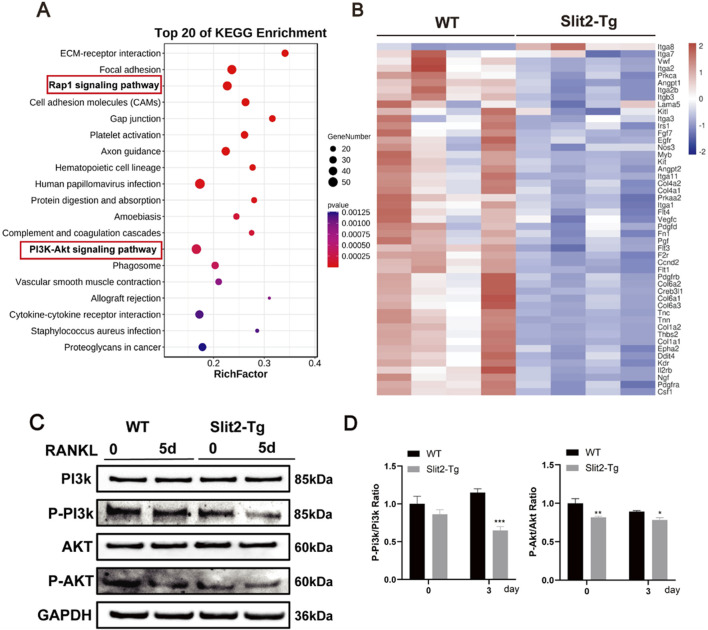
Slit2 overexpression inhibits osteoclast-related signaling pathways. **(A)** High-throughput sequencing KEGG pathway enrichment analysis. **(B)** Heatmap analysis of differential genes associated with PI3K-Akt pathway. **(C)** Western blot analysis of PI3K, AKT, and their phosphorylation levels after 5 days of osteoclast differentiation in BMMs from 8-week-old WT and Slit2-Tg mice (n = 3). **(D)** Quantification of Western blot results. Statistical significance: *p < 0.05, **p < 0.01, ***p < 0.001.

Previous studies demonstrated that Slit2-Tg mice exhibited improved bone quality, but the underlying mechanisms remained unclear. To investigate this, BMMs from WT and Slit2-Tg mice were isolated and induced to differentiate into osteoclasts, followed by high-throughput sequencing analysis. The results identified 1,500 differentially expressed genes, of which 1,300 were downregulated and 200 were upregulated. KEGG enrichment analysis identified the top 20 significantly enriched pathways (Figure 5A), with both Rap1 and PI3K/Akt signaling pathways showing marked suppression in Slit2-Tg mice versus WT controls. Heatmap visualization of PI3K/Akt pathway genes demonstrated significant downregulation of 85% PI3K-associated genes in Slit2-Tg-derived BMMs post-osteoclastogenic induction (Figure 5B). To further validate the expression levels of key signaling pathways in both groups, Western blot analysis was performed. The results confirmed that, compared to WT mice, macrophages derived from Slit2-Tg mice exhibited significantly lower phosphorylation levels of PI3K and Akt proteins following RANKL stimulation for 5 days (Figures 5C,D). These findings suggest that Slit2 may regulate osteoclast differentiation in macrophages by inhibiting PI3K/Akt phosphorylation.

### 3.6 Src expression is reduced in femur bone and cells of Slit2-Tg mice, while Src activation rescues PI3K phosphorylation levels

**FIGURE 6:**
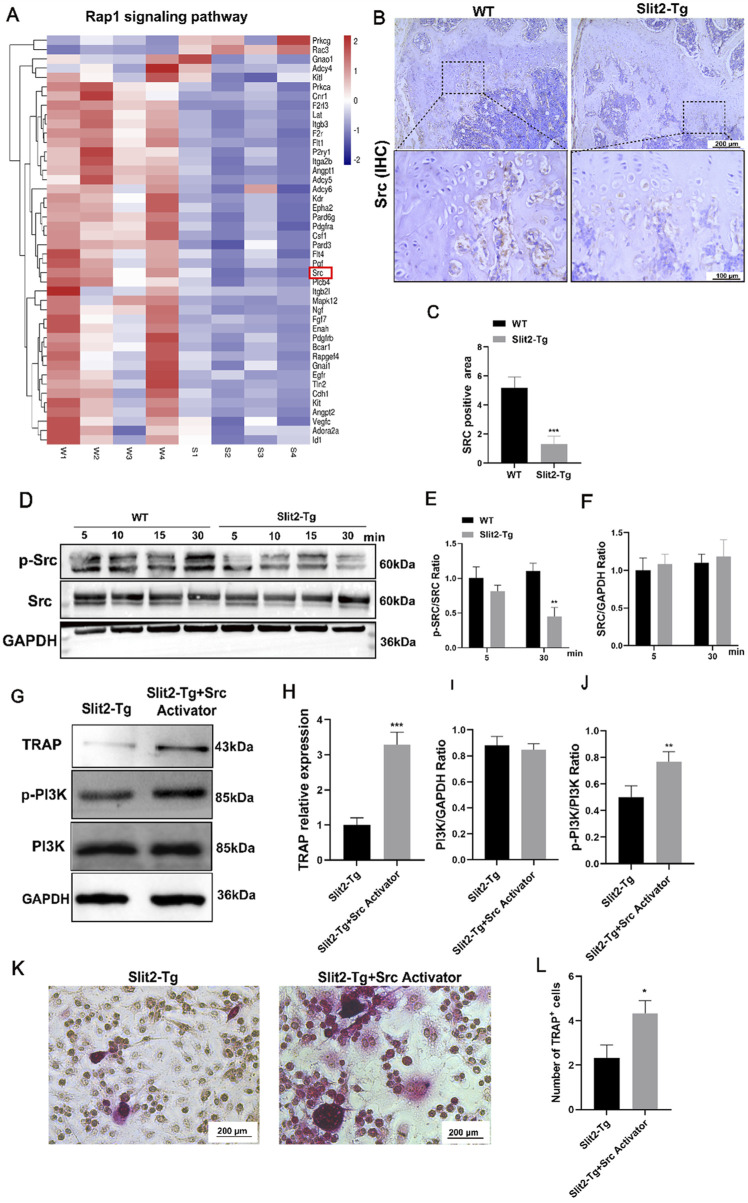
Reduced Src expression in the femoral bone and cells of Slit2-Tg mice, with osteoclast differentiation and PI3K phosphorylation levels restored upon Src activation. **(A)** Heatmap analysis of Rap1 pathway-related differential genes. **(B)** Src immunohistochemical staining results of femur sections of WT mice and Slit2-Tg mice with a scale of 200 μm and 100 μm. **(C)** Statistics of Src immunohistochemical staining of femur sections of 8-week-old WT mice and Slit2-Tg mice (n = 3). **(D–F)** Expression of SRC and P-Src protein in BMMs of WT mice and Slit2-Tg mice after osteoclast differentiation induction using M-CSF and RANKL and M-CSF media (n = 3). **(G)** Expression of TRAP, PI3K and its phosphorylation in BMMs derived from Slit2-Tg mice after adding Src activator (n = 3). **(H–J)** TRAP, PI3K and its phosphorylation. **(K)** TRAP staining results. **(L)** TRAP staining statistics of BMMs derived from Slit2-Tg mice after adding Src activator (n = 3). Data are presented as mean ± SD. The significant difference among the groups, *p < 0.05, **p < 0.01, ***p < 0.001.

To validate the results of the transcriptome sequencing analysis, we analyzed the upstream and downstream pathways of PI3K and found that Src, a key component of the Rap1 signaling pathway, is associated with osteoclast-related signaling. Transcriptomic data revealed that Src expression was significantly suppressed in Slit2-Tg mice (Figure 6A). To further confirm this finding, we performed immunohistochemical (IHC) staining for Src in femoral sections from WT and Slit2-Tg mice. The results demonstrated that Src expression was markedly reduced in the femoral metaphysis of Slit2-Tg mice, which was consistent with the transcriptomic analysis and predicted outcomes (Figures 6B,C). Next, we isolated BMMs from 8-week-old WT and Slit2-Tg mice, and after osteoclast differentiation induction, performed Western blot analysis to validate the expression of key signaling components (Figures 6D–F). These findings suggest that Slit2 may influence osteoclast differentiation in BMMs by inhibiting Src phosphorylation. To further determine whether Slit2 regulates BMMs osteoclast differentiation through Src signaling, a rescue experiment was conducted using an Src activator. BMMs from Slit2-Tg mice were treated with the Src activator (Tolimidone, 10 uM, 24 h) during osteoclast differentiation, and the effects on signaling pathways were assessed. The Western blotting results from the rescue experiments demonstrated that SRC activator-treated BMMs exhibited upregulated significantly expression of the osteoclast-associated factor TRAP and restored PI3K phosphorylation levels (Figures 6G–J). Concurrently, TRAP staining revealed that compared to the SRC agonist-treated BMMs group, osteoclasts derived from Slit2-Tg mice displayed significant suppression at the cell fusion stage, characterized by smaller morphological features and reduced quantities (Figures 6K,L). These findings collectively indicate that BMMs from the Slit2-Tg group suppress SRC expression, inhibit PI3K pathway activation, and thereby impair osteoclast differentiation.

## 4 Discussion

**FIGURE 7:**
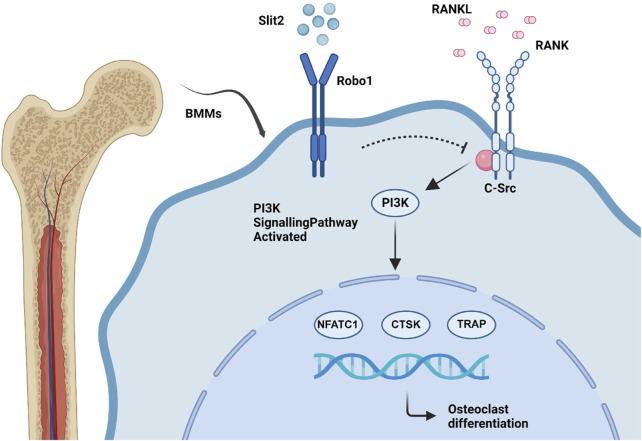
Scheme showing the role of SLIT2 overexpression in macrophage osteoclast differentiation.

Osteoporosis is a prevalent systemic skeletal disease characterized by reduced bone mass and deterioration of bone microarchitecture. Its underlying pathogenesis is primarily driven by excessive osteoclast-mediated bone resorption (Zur et al., 2018; Liu et al., 2023). In this study, we demonstrated that Slit2-Tg mice exhibit superior femur bone properties compared to WT controls in both aging and ovariectomy-induced osteoporosis models. *In vitro* osteoclast differentiation assays, we observed that Slit2 regulated the expression of osteoclast-associated markers and suppressed the differentiation of BMM-derived osteoclasts. Mechanistically, Slit2 inhibited osteoclast differentiation by suppressing the PI3K signaling pathway. Further analysis of the Rap1 signaling pathway identified Src as a key factor, and subsequent experiments confirmed that Src plays a crucial role in Slit2-mediated inhibition of the PI3K pathway. Notably, treatment with Src activator restored PI3K phosphorylation levels (Figure 7). Our findings suggest that elevated Slit2 levels in bone tissue may exert a protective effect against osteoporosis progression, providing valuable insights into the potential of Slit2 as a therapeutic target for osteoporosis.

In recent years, an increasing number of studies have demonstrated that neural axon guidance molecules, including Slit, Sema, and Netrins, play critical roles in bone regeneration and resorption (Negishi-Koga et al., 2011; Zhu et al., 2019; Chen et al., 2020)^.^ In this study, Micro-CT and H&E staining analyses revealed that the femoral microarchitecture in both aging-induced and ovariectomy-induced osteoporosis models of Slit2-Tg mice were significantly superior to that of WT mice. Notably, the Slit2-Tg group demonstrated superior bone mass phenotypes relative to WT mice even in control model cohorts. These consistent findings across different age groups and osteoporosis models suggest that Slit2 plays a critical role in bone metabolism regulation and may have broad applicability in osteoporosis pathogenesis. Furthermore, we observed that serum CTX1 levels were lower in Slit2-Tg mice compared to WT mice. As a key biomarker of bone resorption, CTX1 is released into circulation following osteoclast-mediated bone resorption, with its circulating concentration directly reflecting systemic osteoclastic capacity. These findings collectively suggest that Slit2 may exert a negative regulatory role in the pathogenesis of OP.

RANKL serves as a critical mediator in osteoclast metabolism and differentiation, driving bone resorption through its capacity to induce osteoclastogenesis of BMMs (Zhang et al., 2022). Studies have reported that Slit2 can reduce macrophage recruitment and infiltration, and negatively regulates cellular migration by inhibiting cytoskeletal remodeling in macrophages (Bhosle et al., 2020; Li et al., 2020). These results suggest that Slit2 may be a crucial factor influencing macrophage osteoclast differentiation and osteoclast number. In our *in vitro* experiments, TRAP staining and immunofluorescence analyses demonstrated that BMMs isolated from Slit2-Tg mice and cultured in osteoclastogenic medium containing M-CSF and receptor activator of RANKL for 5 days exhibited significantly fewer osteoclasts number and smaller multinucleated cell sizes compared to wild-type controls. Similarly, RT-qPCR and Western blot analyses demonstrated that the expression levels of osteoclast-related markers, including *Ctsk*/CTSK and *Nfatc1*/NFACT1, were markedly lower in Slit2-Tg-derived BMMs than in WT BMMs. These molecular findings further indicate that Slit2 may disrupt a critical step in osteoclastogenesis, leading to impaired osteoclast development, which ultimately contributes to the enhanced bone microarchitecture observed in Slit2-Tg mice.

The PI3K/Akt signaling pathway is closely associated with osteoclast proliferation and differentiation (Wang et al., 2019; Zhong et al., 2019). High-throughput sequencing of osteoclasts from Slit2-Tg mice revealed that both the PI3K/Akt and Rap1 signaling pathways were suppressed. Previous studies have shown that when insulin-like growth factor (IGF) activates PI3K, the subsequent activation of Akt leads to the phosphorylation of serine residues on GSK3β, thereby inhibiting NFATc1 expression and preventing the fusion of osteoclast precursors (Xiao et al., 2020). This finding suggests that the PI3K/Akt signaling pathway plays a role in regulating osteoclast precursor fusion, ultimately affecting osteoclastogenesis. However, a potential mechanistic limitation in our current study that necessitates future *in vitro* validation of Slit2’s effects on osteoclast precursors. Our analysis of differentially regulated pathways identified Src involvement. As an upstream regulator of the PI3K signaling pathway, the Rap1 signaling pathway plays a crucial role in cell adhesion and intercellular connections, making it essential for the fusion of mononuclear macrophages into mature osteoclasts. As a key member of the Rap1 pathway, Src has been reported to attenuate RANKL-induced osteoclast differentiation via the c-Src/PI3K pathway (Zhou et al., 2020; Luo et al., 2021). Immunohistochemistry and Western blot analyses of mouse femoral sections confirmed that Src expression was downregulated in response to Slit2 overexpression, indicating a negative regulatory relationship between Src and Slit2 expression. Furthermore, upon Src activation, an increase in PI3K phosphorylation levels was observed, suggesting that Slit2 inhibits osteoclast differentiation of macrophages via the Src/PI3K signaling axis. However, Future experiments should include TRAP staining and analyzing osteoclast-related signaling pathways after Src activator treatment, to confirm that Slit2 inhibits osteoclast differentiation by downregulating PI3K.

In addition to these findings, transcriptomic profiling further revealed that Slit2 exerts widespread regulatory effects on multiple signaling pathways in BMMs, including Rap1, MAPK, and PI3K-AKT, which are crucial for osteoclast precursor adhesion, fusion, and activation. Previous studies have reported that the Rap1 pathway enhances integrin activity and promotes cytoskeletal polarization essential for osteoclast maturation and function (Teitelbaum, 2000). Our data suggest that Slit2 may indirectly inhibit the PI3K/AKT axis by downregulating the Rap1–Src signaling cascade, thereby impairing osteoclastogenesis at multiple levels.

Interestingly, despite its profound effect on bone resorption, Slit2 did not enhance osteogenic differentiation, as indicated by unaltered ALP activity in bone marrow stromal cells. This suggests that Slit2’s osteoprotective effects are primarily mediated through suppression of osteoclast activity rather than direct stimulation of osteoblast function. This observation is consistent with previous findings by Park et al., who showed that Slit2 impairs osteoclast formation by inhibiting Cdc42 activity without affecting osteoblasts (Park et al., 2019).

From an immunomodulatory perspective, Slit2 belongs to a class of axon guidance molecules increasingly recognized for their roles in immune regulation. It has been shown to suppress macrophage migration, infiltration, and actin remodeling, thereby affecting inflammatory responses in various disease models (Teitelbaum, 2000; Bhosle et al., 2020; Li et al., 2020). In our study, the significant downregulation of Src expression and phosphorylation in Slit2-Tg BMMs underscores Slit2’s upstream regulatory influence on osteoclastogenic signaling and supports its dual role as a neuro-immune–skeletal mediator.

Despite these promising results, our study has limitations. Whether Slit2 interferes with early RANK–RANKL signaling events or modulates upstream mediators like TRAF6 and NF-κB remains to be elucidated. Future studies using Slit2 knockout models or exogenous recombinant Slit2 protein supplementation will help clarify its full therapeutic potential in osteoporosis.

## 5 Conclusion

This study presents the first comprehensive elucidation of Slit2’s regulatory mechanism in osteoporotic bone remodeling. Using both aging-induced and ovariectomy-induced osteoporosis models in wild-type and Slit2-transgenic mice, we demonstrate that Slit2 overexpression markedly mitigates bone loss and preserves trabecular architecture. *In vitro* assays of bone marrow-derived macrophages reveal that Slit2 attenuates osteoclastogenesis, as evidenced by reduced TRAP-positive multinucleated cells and downregulation of osteoclast markers such as NFATc1 and CTSK. Mechanistic investigations—including RNA sequencing, GO pathway enrichment, and Western blot analyses—identify the Src-PI3K/AKT signaling axis as the critical mediator: Slit2 suppresses Src expression and inhibits downstream PI3K and AKT phosphorylation, thereby impairing osteoclast differentiation and bone resorption. Decreased serum CTX-1 levels in Slit2-Tg mice further corroborate the inhibition of bone resorption. Collectively, these findings highlight Slit2 as a promising therapeutic target for osteoporosis.

## Data Availability

The data that support the findings of this study are available from the corresponding author upon reasonable request.
